# Pharmacokinetic and pharmacodynamic properties of polymyxin B in *Escherichia coli* and *Klebsiella pneumoniae* murine infection models

**DOI:** 10.1093/jac/dkad022

**Published:** 2023-01-30

**Authors:** Aart van der Meijden, Vincent Aranzana-Climent, Heleen van der Spek, Brenda C M de Winter, William Couet, Joseph Meletiadis, Anouk E Muller, Sanne van den Berg

**Affiliations:** Department of Medical Microbiology and Infectious Diseases, Erasmus MC, University Medical Center, Rotterdam, The Netherlands; INSERM U1070, CHU de Poitiers et Université de Poitiers, Poitiers, France; Department of Medical Microbiology and Infectious Diseases, Erasmus MC, University Medical Center, Rotterdam, The Netherlands; Department of Hospital Pharmacy, Erasmus MC, University Medical Center, Rotterdam, The Netherlands; CATOR, Center for Antimicrobial Treatment Optimization Rotterdam, Rotterdam, The Netherlands; Rotterdam Clinical Pharmacometrics Group, Rotterdam, The Netherlands; INSERM U1070, CHU de Poitiers et Université de Poitiers, Poitiers, France; Department of Medical Microbiology and Infectious Diseases, Erasmus MC, University Medical Center, Rotterdam, The Netherlands; Clinical Microbiology Laboratory, Attikon University General Hospital, Medical School, National and Kapodistrian University of Athens, Athens, Greece; Department of Medical Microbiology and Infectious Diseases, Erasmus MC, University Medical Center, Rotterdam, The Netherlands; CATOR, Center for Antimicrobial Treatment Optimization Rotterdam, Rotterdam, The Netherlands; Department of Medical Microbiology, Haaglanden MC, The Hague, The Netherlands; Department of Medical Microbiology and Infectious Diseases, Erasmus MC, University Medical Center, Rotterdam, The Netherlands; CATOR, Center for Antimicrobial Treatment Optimization Rotterdam, Rotterdam, The Netherlands

## Abstract

**Background:**

Although polymyxin B has been in use since the late 1950s, there have been limited studies done to unravel its pharmacokinetics (PK) and pharmacodynamics (PD) index.

**Methods:**

We determined, in neutropenic infected mice, the PK, plasma protein binding and PK/PD index best correlating with efficacy for *Escherichia coli* and *Klebsiella pneumoniae* strains.

**Results:**

The pharmacokinetic profile showed non-linear PK; dose was significantly correlated with absorption rate and clearance. The inhibitory sigmoid dose–effect model for the *fC*_max_/MIC index of *E. coli* fitted best, but was only modestly higher than the R^2^ of *%fT_>_*_MIC_ and *f*AUC/MIC (R^2^ 0.91–0.93). For *K. pneumoniae* the *f*AUC/MIC index had the best fit, which was slightly higher than the R^2^ of *%fT_>_*_MIC_ and *fC*_max_/MIC (R^2^ 0.85–0.91). Static targets of polymyxin B *f*AUC/MIC were 27.5–102.6 (median 63.5) and 5.9–60.5 (median 11.6) in *E. coli* and in *K. pneumoniae* isolates, respectively. A 1 log kill effect was only reached in two *E. coli* isolates and one *K. pneumoniae*. The PTA with the standard dosing was low for isolates with MIC >0.25 mg/L.

**Conclusions:**

This study confirms that *f*AUC/MIC can describe the exposure–response relationship for polymyxin B. The 1 log kill effect was achieved in the minority of the isolates whereas polymyxin B PK/PD targets cannot be attained for the majority of clinical isolates with the standard dosing regimen, indicating that polymyxin B may be not effective against serious infections as monotherapy.

## Introduction

With the emergence of resistance and limited therapeutic options, the use of neglected and disused antibiotics becomes increasingly important. However, knowledge about pharmacokinetics (PK) and pharmacodynamics (PD) of these old antibiotics is mostly lacking or very limited. Polymyxin B is such an antibiotic, with activity against bacteria belonging to the largest antibiotic resistance threats according to the US CDC,^1^ including carbapenem-resistant Enterobacterales, *Pseudomonas aeruginosa* and *Acinetobacter baumannii*.

The use of polymyxin B fell out of favour in the mid-1970s, because of toxicity concerns, especially regarding nephrotoxicity and neurotoxicity,^2^ variability in exposure profiles and the narrow therapeutic window.^3^ Nowadays, it is increasingly used as a last-line antibiotic. The somewhat simpler PK of polymyxin B, compared with colistin, might be a therapeutic advantage, but nevertheless polymyxin B is currently not available in the EU.

Limited studies have investigated the PK/PD of polymyxin B, so optimal dosing regimens remain uncertain.^3,4^ Earlier *in vitro* and *in vivo* studies mainly focused on colistin PD in *P. aeruginosa* and *A. baumannii* isolates.^5–7^ In 2017, Landersdorfer *et al.*^8^ were the first to show in studies in mice that polymyxin B antimicrobial activity against *Klebsiella pneumoniae* best correlated with the ratio of the area under the unbound concentration–time curve to the MIC (*f*AUC/MIC). In addition, other *in vitro* studies using various Gram-negative species have suggested *f*AUC/MIC is relevant for polymyxin B,^9,10^ which is in line with results for colistin.^11^

In the present study, we investigated which PK/PD index is best correlated with efficacy of an *Escherichia coli* and a *K. pneumoniae* strain. Subsequently, we determined the magnitude of the PK/PD index needed for stasis and a 1 log kill in several *E. coli* and *K. pneumoniae* strains.

## Materials and methods

### Antibiotics and bacterial strains

Polymyxin B sulphate salt (lot. 117M4045V, 8926 units/mg; Sigma–Aldrich, Zwijndrecht, The Netherlands) and was reconstituted in normal sterile saline (0.9% NaCl, Baxter, Utrecht, The Netherlands). Solutions were freshly prepared for each experiment.

Four *E. coli* and five *K. pneumoniae* well-characterized clinical isolates with different resistance mechanisms were used (Table S1, available as Supplementary data at *JAC* Online).

### In vitro susceptibility testing

The MIC of polymyxin B for each strain was determined in triplicate by broth microdilution, according to the CLSI guidelines.^12,13^ Median MIC values were reported and utilized in the PK/PD analysis.

### In vivo thigh and lung infection models in neutropenic mice

Experiments were carried out in the Erasmus Laboratory Animal Science Centre in Rotterdam, The Netherlands in accordance with the EU Animal Directive 2010/63/EU 2010 directive^14^ (licence number AVD101002016702) as described before,^15^ with approval of the institutional Animal Welfare Body. Outbred female CD-1 mice obtained from Charles River Germany, weighing 20–25 g, 7–8 weeks old, were used in the experiments. The mice were housed under standard conditions with drink and feed supplied *ad libitum.*

Mice were rendered neutropenic by intraperitoneal injection of cyclophosphamide of 150 and 100 mg/kg, 4 days and 1 day before infection, respectively. On the day of infection, an overnight culture of the bacteria was diluted in fresh CAMHB and incubated at 37°C for 1 or 2 h depending on the growth rate of the strain. After incubation the inoculum was diluted in CAMHB or saline for the thigh and lung infection model, respectively, in order to obtain approximately 5.0 × 10^7^ cfu/mL. Mice were then infected intramuscularly in each thigh (thigh infection model) or intranasally under isoflurane anaesthesia (lung infection model), with 0.05 mL of inoculum containing approximately 2.5 × 10^6^ bacteria. Analgesia was given in the form of buprenorphine directly after infection, every 12 h.

### PK of polymyxin B

Mice were injected subcutaneously with a 0.1 mL single dose of polymyxin B 2 h after infection (*t* = 0). The PK of polymyxin B ranging between 0.5 and 64 mg/kg were determined in two mice per dose level. Mice were separated into two groups, with four dose levels studied in the thigh infection model (0.5, 2, 8, 32 mg/kg) and four dose levels studied in the lung infection model (1, 4, 16, 64 mg/kg). At *t* = 0, two mice were humanely euthanized to determine the bacterial load in the infected organ at the start of infection. At 10 different timepoints, blood was collected under isoflurane anaesthesia, in K3E EDTA tubes (Sarstedt, Nümbracht, Germany) through orbital sinus bleeding, which was immediately followed by cervical dislocation. The collected blood samples were centrifuged immediately at 15 871 RCF for 5 min at 4°C. Plasma was stored at −80°C until analysis.

Bronchoalveolar lavage (BAL) was performed immediately after blood collection. The trachea was exposed by a ventral vertical incision in the neck for insertion of a cannula. Lungs were instilled two times with 1 mL of sterile saline, and the fluid was recovered immediately. The recovered lavages were pooled, directly placed on ice, and subsequently stored at −80°C.

Since the PK were analysed using a population PK model, which increases the power of the analysis, we used two mice per timepoint to detect outliers.

### Protein binding

The binding of polymyxin B to plasma proteins was determined via ultrafiltration. Polymyxins have a tendency to non-specifically bind to different kinds of material, including ultrafiltration filters. Thus, to correct for polymyxin B loss on the filter, an experiment was performed in which ultrafiltrated (UF) phosphate buffer was spiked with multiple polymyxin B concentrations (ranging from 0.75 to 8 mg/L) and subsequently concentrations were measured. From these results the relationship between polymyxin B concentration post-UF and non-specific binding could be established.^16^ Using this relationship, the non-specific binding (NSB) for each measured polymyxin B UF concentration was calculated. Correction for the measured polymyxin B UF concentration using this NSB value was according to the following formula: polymyxin B UF Corrected Concentration = Measured polymyxin B UF Concentration × (1−NSB).

### Epithelial lining fluid (ELF) concentration

By taking BAL fluid samples and a plasma sample at the same timepoint, the concentrations in ELF could be determined. The ratio of the urea concentration in BAL to the concentration in plasma was used to determine the ELF concentration.^17^ In short, polymyxin B concentration in ELF was calculated by multiplying the polymyxin B concentration in the BAL fluid by the ratio of the urea concentration in the plasma and BAL fluid: [polymyxin B]_ELF_ = [polymyxin B]_BAL_ × ([urea]_Plasma_/[urea]_BAL_). Urea concentrations were measured with an enzymatic assay (QuantiChrom urea assay kit; DIUR-100; BioAssay Systems, Hayward, CA, USA).

### LC-MS/MS assay

Polymyxin B concentrations in plasma, BAL and ultrafiltrates were determined by a validated LC-MS/MS method,^18^ with a lower limit of quantification (LOQ) of 0.2 mg/L for plasma and 0.05 mg/L for BAL fluid and ultrafiltrates. In this assay, 0.1 mL of plasma or 0.05 mL of BAL fluid or ultrafiltrates was added to 0.05 mL of drug-free plasma, and was mixed with 0.75 mL of phosphate buffer (pH 7.2) and 0.01 mL of internal standard (colistin sulphate, Merck KGaA, Darmstadt, Germany) at 6.25 mg/L for plasma and 5 mg/L for BAL fluid and ultrafiltrates. The samples were briefly vortexed and then centrifuged at 3000 rpm for 5 min. The supernatants (0.8 mL) were loaded onto solid-phase extraction columns (Oasis HLB solid-phase extraction cartridges, 1 mL, 30 mg, Waters, Saint-Quentin-en-Yvelines, France), preconditioned with 1 mL of methanol and followed by 1 mL of water. Columns were washed with 1 mL of water and dried under nitrogen pressure. The analytes were eluted with 0.5% formic acid in methanol and they were evaporated at 45°C under a gentle nitrogen jet stream. The residues were dissolved in 0.1 mL of 0.1% formic acid in water and analysed by LC-MS/MS. The system included an Alliance Waters 2695 LC system module (Waters) coupled with an API Quattro Micro (Waters). Polymyxin B was analysed on an XBridge C_18_ column (5 µm, 2.1 × 150 mm; Waters). The mobile phase A consisted of 0.1% formic acid in water, and mobile phase B was 0.1% formic acid in acetonitrile. The gradient for mobile phases A and B were respectively set at 75% and 25% with a flow rate of 0.2 mL/min. Electrospray ionization in positive mode was used for the detection of polymyxin B. Ions were analysed in the multiple reaction monitoring, and the following transitions were inspected: *m/z* 602.1→101 for polymyxin B1, *m/z* 595.1→101 for polymyxin B2, *m/z* 585.1→101 for CSTA and *m/z* 578.1→101 for CSTB. Calibration curves of polymyxin B ranged from 0.1 to 10 mg/L for plasma and ultrafiltrates, and were quantified with a quadratic regression mode. The intraday variability was characterized at four levels (0.3, 1, 2.5 and 10 mg/L) with a precision and bias of <20% for the lowest level, and <15% for the others.

### Pharmacokinetic modelling

A population PK model was developed using non-linear mixed-effects modelling (NONMEM, version 7.4.2 ICON Development Solutions, Ellicott City, MD, USA). The analysis was performed using the FOCE + I method on logarithmically transformed concentrations and data below the LOQ were omitted. Parameters were calculated for a virtual 1 kg mouse, resulting in pharmacokinetic parameters corresponding on a per kg base. In the first step a structural model was developed, using one- and two-compartment models. The absorption following subcutaneous injections was described using an absorption rate constant (k_a_). Typical values for central (*V*_c_) and peripheral volume of distribution (*V*_p_), CL and intercompartmental clearance (Q) were estimated. As bioavailability (F) could not be estimated, CL, *V*_c_, Q, and *V*_p_ values corresponded to the ratios CL/F, *V*_c_/F, Q/F and *V*_p_/F, respectively. Addition of between-subject variability (BSV), described using an exponential model, was evaluated for each pharmacokinetic parameter. Residual variability was described using an additive error model for logarithmically transformed data. In the second step, relationships between pharmacokinetic parameters and potential covariates (infection site and polymyxin B dose) were investigated. The effect of dose on the parameters was modelled using a power function, effect of infection site with a linear function. Covariates were included using forward inclusion (*P* < 0.05) and backward elimination (*P* < 0.001). Minimum objective function values (OFVs), parameter precision, error estimates and visual inspection of the goodness-of-fit plots were considered for model selection. CIs around the final parameters were estimated by bootstrap with resampling (*n* = 1000) and fit to the data was evaluated using visual predictive checks (VPCs) and VPCs stratified for dose (*n* = 1000).

### PD of polymyxin B

Dose-fractionation studies were performed for *E. coli* ATCC 25922 and *K. pneumoniae* 104 in the thigh infection model. Two hours after infection, treatment with polymyxin B was administered subcutaneously over 24 h at the following dosing regimens: 4–128 mg/kg q6h [total daily dose (TDD) 16–512 mg/kg]; 8–256 mg/kg q12h (TDD 16–512 mg/kg); 256–512 mg/kg q24h (TDD 256–512 mg/kg).

For dose–response studies, a 6 hourly dosing regimen of 1–32 mg/kg was studied in the thigh infection model. Mice were infected with *E. coli* ATCC 259222, 15, 51 or 71 or *K. pneumoniae* ATCC 43816, 17, 58 or 74.

For PD analysis, we used six doses in two animals, resulting in 12 data points. As a rule of thumb, 2*n* + 1 = 9 data points (*n* = model parameters, which in the case of the E_max_ model is 4; the E_max_, E_min_, ED_50_ and slope) are required in regression analysis.

Animals were humanely euthanized 24 h (*t* = 24 h) after the first dose, unless the welfare of the animals necessitated earlier termination, following animal welfare regulations. Excised thighs were transferred to a pre-cooled 14 mL polypropylene tube containing 2 mL of PBS and were homogenized using a T25 ULTRA-TURRAX (IKA-Werke GmbH & Co, Staufen, Germany). A 10-fold dilution series was prepared and 3 × 10 μL was plated per dilution. The following day, colonies were counted and the number of cfu per thigh was calculated.

The drug effect was determined by the difference between the log_10_ cfu values at *t* = 0 h (mean value of two mice) and *t* = 24 h (value of individual mice) expressed as Δlog_10_ cfu. The *f*AUC/MIC, *f*C**_max_/MIC and %*fT_>_*_MIC_ were determined based on the simulated time–concentration profiles over 24 h as derived in MicLab 2.71 (MEDIMATICS, Maastricht, The Netherlands) using 200 points per dosing interval.

A sigmoid maximum effect (E_max_) model was fitted to the PK/PD index versus Δlog_10_ cfu data by non-linear regression, and static or a 1 log or 2 log kill effect were determined using GraphPad Prism 8.0 (GraphPad, Inc., San Diego, CA, USA). A two-tailed unpaired *t*-test was performed in GraphPad Prism 8.0 to determine if there was a significant difference in the *f*AUC/MIC targets correlating with a static or a 1 log or 2 log decline, between *E. coli* and *K. pneumoniae* isolates.

### PTA in humans

A previously published population PK model^19^ and Monte Carlo simulations was used to estimate the *f*AUCs of 5000 patients treated with 200 mg q24h (MicLab 2.71) taking into account protein-binding rates ranging from 36% to 98% as previously reported.^20–23^ The PTA was calculated for isolates with MICs ranging from 0.25 to 4 mg/L.

## Results

### Plasma protein binding

NSB was concentration dependent and log-linearly correlated with concentrations ranging from 77% ± 8.3% at 0.75 mg/L to 20% ± 0.6% at 8 mg/L. No concentration dependency was observed for polymyxin B and %f_u_ (percent fraction unbound; mean ± SD) was determined to be 20% ± 4.0%. For the calculation of exposures to unbound compound [maximum concentration in plasma of the free fraction of drug (*fC*_max_)] and *f*AUC, a single factor of 20% was used.

### PK

**Figure 1.**
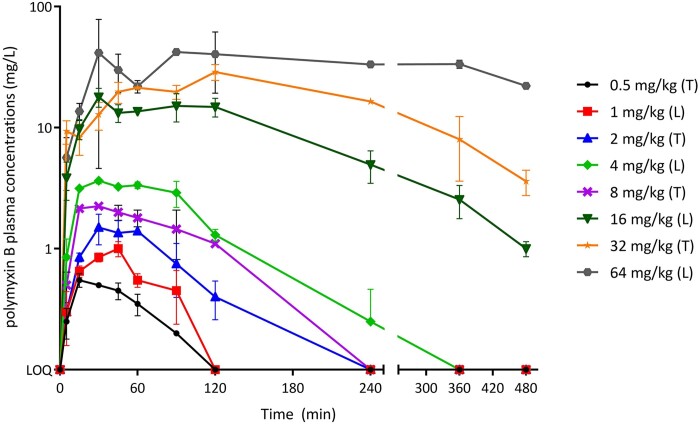
Total plasma polymyxin B concentration versus time after single subcutaneous administrations of 2, 4, 8, 16, 32 or 64 mg/kg polymyxin B in infected neutropenic mice. Each data point represents two mice, with the mean ± SD plotted. Lower LOQ is 0.2 mg/L. T, thigh infection model; L, lung infection model. This figure appears in colour in the online version of *JAC* and in black and white in the print version of *JAC*.

The pharmacokinetic profile of polymyxin B in mouse plasma after single subcutaneous administrations of 0.5–64 mg/kg is shown in Figure 1. The total peak plasma levels ranged from 0.6 to 68 mg/L, with detectable polymyxin B plasma levels up to 6 and 8 h after subcutaneous administration for doses up to 8 and 16–64 mg/kg, respectively. Low levels of polymyxin B in ELF were only detectable after a 64 mg/kg dose (data not shown); other concentrations in ELF were below the LOQ.

Whatever the infection model, time to peak (*T*_max_) was ≤2 h. There were no significant differences in PK between thigh and lung models (Figure S1).

### Pharmacokinetic modelling

The PK profile was described by a one-compartment model with first-order absorption. Inclusion of BSV on k_a_ and *V*_c_ improved the model fit. CL and k_a_ were significantly correlated with polymyxin B dose (Table 1 and Figure S2).

**Table 1.** Parameter estimates and bootstrap analysis of the population PK model for polymyxin B

Parameter	Description	Value	Bootstrap (5^th^/95^th^ percentile)
k_a_ (h^−1^)	Absorption rate constant for 10 mg/kg dose	1.63	1.25/2.20
*V*_c_/F (L/kg)	Central volume of distribution	0.774	0.669/0.880
CL/F (L/h/kg)	Clearance for 10 mg/kg dose	0.384	0.348/0.424
Dose on k_a_	Power on covariate effect of dose on k_a_	−0.407	−0.485/−0.3217
Dose on CL	Power on covariate effect of dose on CL	−0.26	−0.322/−0.197
BSV k_a_ (%)	Inter individual variability on k_a_	79	64/96
BSV CL (%)	Inter-individual variability on clearance	45	20/58
Residual error	Variance of the log additive residual error	0.093	0.040/0.167

The covariate polymyxin B dose (in mg/kg) is added as follows: CL = 0.384 × (DOSE/10)^−0.26^ and k_a _= 1.63 × (DOSE/10)^−0.407^. Bootstrap is presented as 90-percentile range of 475 successful resampling runs.

The goodness-of-fit plots showed good agreement between observations and model predictions. The final model was validated using bootstrap and VPC (Figure S3). No effect on PK was found for infection site.

In the PK model the dose per administration is a covariate on CL as well as k_a_, which means that total drug exposure of the same TDDs differs between the q6h and q12h intervals.

### Dose fractionation study

**Figure 2.**
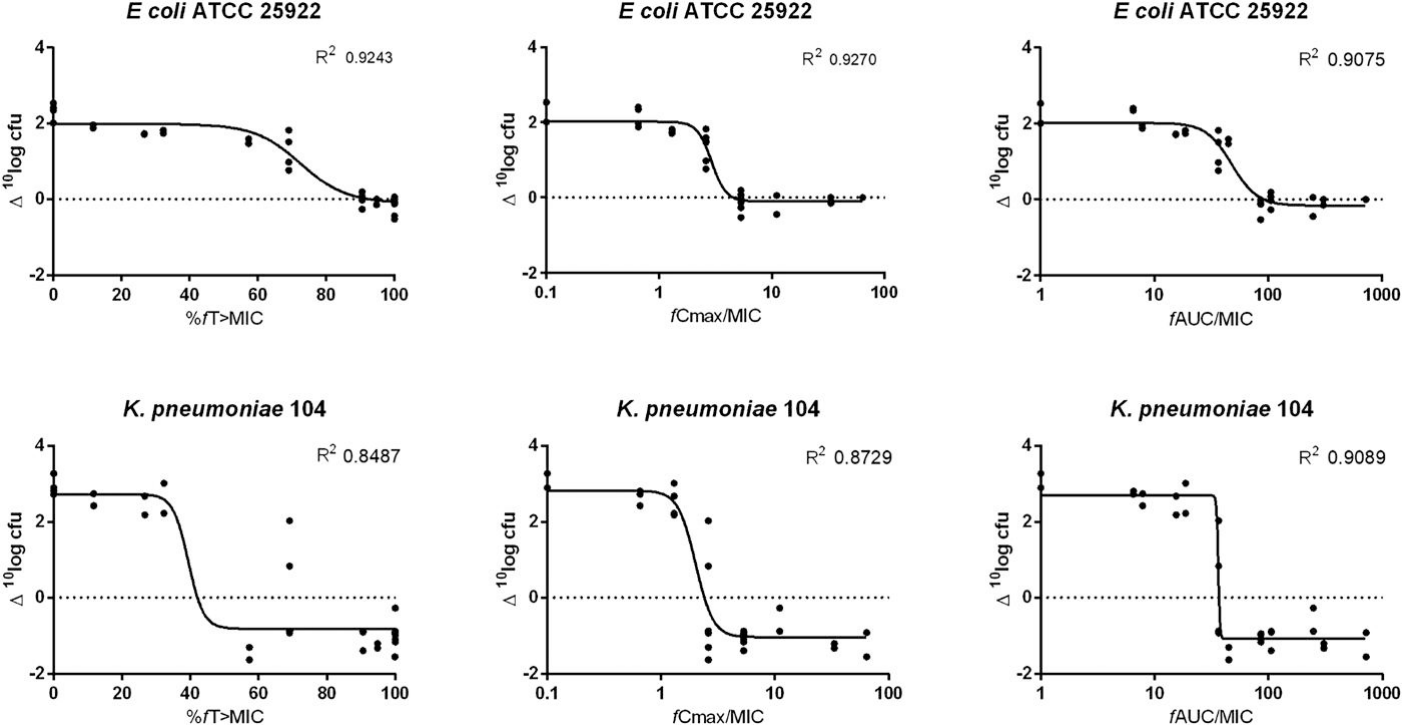
Dose-fractionation studies. Relationships after polymyxin B treatment, where the bacterial burden, expressed as Δlog_10_ cfu, is plotted against %*fT_>_*_MIC_, *fC*_max_/MIC and *f*AUC/MIC for *E. coli* ATCC 25922 and *K. pneumoniae* 104. Each data point represents a single thigh; the solid line represents the fit of the inhibitory sigmoid dose–effect model; the dotted line indicates the point at which there is no change from the burden at the start of treatment.

In dose fractionation studies the average bacterial burden at start of treatment (*t* = 0) was 1.5 × 10^7^ (range: 1.2–2.0 × 10^7^) cfu/thigh. Polymyxin B doses >64 mg/kg were not tolerated. The relationship between the bacterial load and *f*AUC/MIC, *fC*_max_/MIC and %*fT_>_*_MIC_ for *E. coli* ATCC 25922 and *K. pneumoniae* 104 is shown in Figure 2. The inhibitory sigmoid dose–effect model for *fC*_max_/MIC of *E. coli* fitted best (R^2^ 0.9270), but was only modestly higher than %*fT_>_*_MIC_ (R^2^ 0.9243) and *f*AUC/MIC (R^2^ 0.9075). For *K. pneumoniae* 104, *f*AUC/MIC had the best fit (R^2^ 0.9089), which was slightly higher than the R^2^ values of 0.8729 for *fC*_max_/MIC and 0.8487 for %*fT_>_*_MIC_.

### Determination of the PK/PD index linked to efficacy

**Figure 3.**
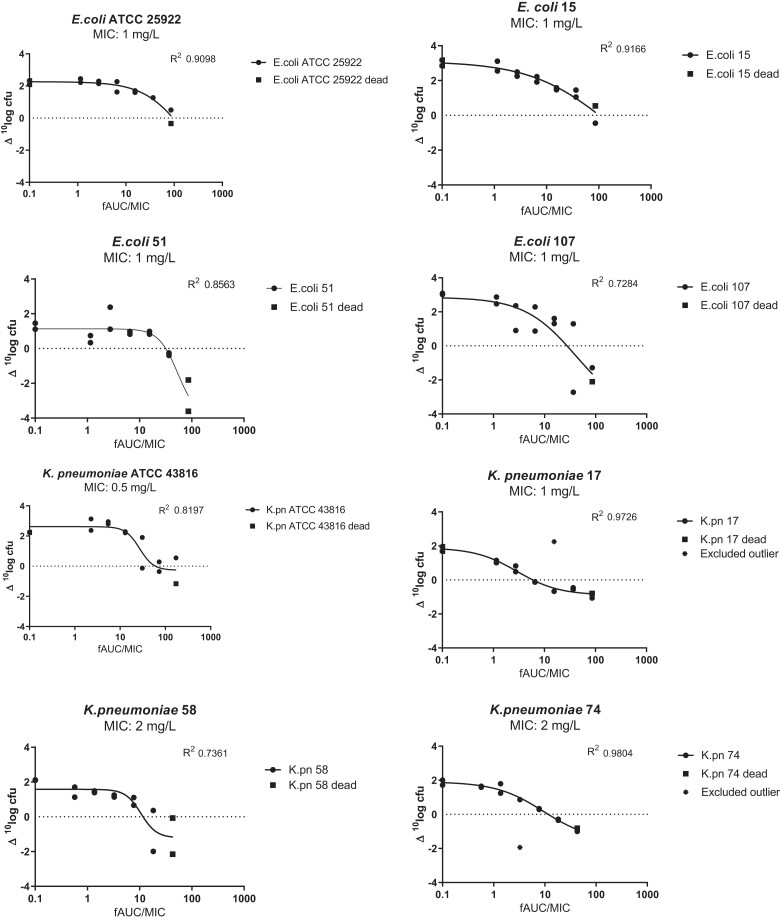
Relationship between the bacterial load in the thighs, expressed as Δlog_10_ cfu, and the *f*AUC/MIC after 24 h of polymyxin B treatment in a 6 hourly dosing interval in the neutropenic mouse thigh infection model for four *E. coli* and four *K. pneumoniae* strains. Each data point represents a single thigh; the solid line represents the fit of the inhibitory sigmoid dose–effect model; the dotted line indicates the point at which there is no change from the burden at the start of treatment.

The dose–response curves for four *E. coli* and four *K. pneumoniae* strains with q6h dosing regimens are shown in Figure 3. At start of treatment, the average bacterial load was 1.5 × 10^7^ (range: 4.0 × 10^6^–3.3 × 10^7^) cfu/thigh. A static effect was achieved in 5/8 isolates (static effect was observed in 1/2 mice for *E. coli* ATCC 25922 and 15, and *K. pneumoniae* ATCC 43816) whereas a 1 log kill effect was observed in 3/8 isolates (*E. coli* 51 and 107 and *K. pneumoniae* 58), and 2 log kill effect in 1/8 isolates (*E. coli* 51). The exposure–response relationships of the eight strains were well described by the E_max_ model (R^2^ > 0.7) and static, 1 log and 2 log cfu reduction effects were calculated. The *f*AUC/MIC correlating with a static, 1 log and 2 log kill effect for the individual strains is presented in Table 2. The *f*AUC/MIC correlating with a static effect in the thigh infection model was not statistically different for the *E. coli* compared with the *K. pneumoniae* strains (the two-tailed *P* value equals 0.1294; unpaired *t*-test). In mice that received the highest dosing regimen, 32 mg/kg q6h (TDD 128 mg/kg), the last (fourth) administration of polymyxin B was not tolerated in 2/4 mice.

**Table 2.** Target values of *f*AUC/MIC for stasis, 1 and 2 log decline in cfu in the neutropenic *E. coli* and *K. pneumoniae* thigh infection model for polymyxin B

				Target values of *f*AUC/MIC		
Bacterial isolate	MIC (mg/L) or data measure	Bacterial burden at start of therapy (log_10_ cfu/thigh)^a^	Increase in bacterial burden at 24 h in untreated control animals (Δlog_10_ cfu/thigh)^a^	static effect	1 log kill	2 log kill
*E coli* ATCC 25922	1	7.6	2.2	95.3	NA	NA
*E. coli* 15	1	7.6	3.0	102.6	NA	NA
*E. coli* 51	1	6.8	1.3	31.6	46.8	65.3
*E. coli* 107	1	7.1	3.1	27.5	54.5	NA
*K. pneumoniae* ATCC 43816	0.5	7.1	2.2	60.5	NA	NA
*K. pneumoniae* 17	1	7.2	1.8	5.9	NA	NA
*K. pneumoniae* 58	2	7.0	2.1	11.6	25.2	NA
*K. pneumoniae* 74	2	7.1	1.9	11.6	NA	NA
*E. coli*	average	7.3	2.4	64.3	—	—
	median	7.3	2.6	63.5	—	—
	SD	0.3	0.7	34.8	—	—
*K. pneumoniae*	average	7.1	2.0	22.4	—	—
	median	7.1	2.0	11.6	—	—
	SD	0.04	0.2	22.1	—	—

NA, not achieved.

Mean of two mice.

### PTA in humans

**Figure 4.**
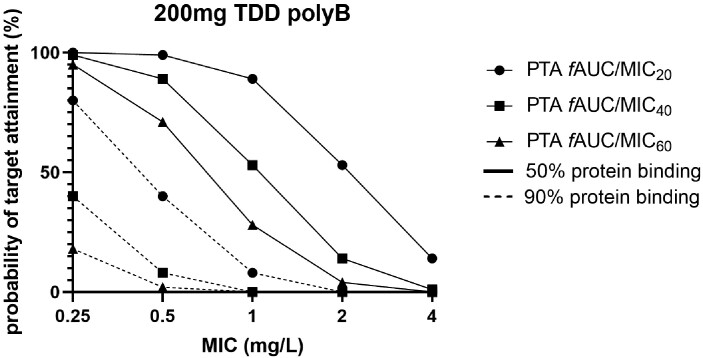
PTA for strains with increasing MICs. Both 50% and 90% protein binding, and *f*AUC/MIC targets of 20, 40 and 60 were taken into account.

As reported values for human protein binding in literature showed a wide range, the PTA for the 200 mg clinical dose q24h (Figure 4) was performed taking into account a protein binding of 50% and 90%. In addition, the PK/PD targets for *f*AUC/MIC of 20, 40 and 60 were analysed in order to include a range for effects from stasis to 2 log kill observed for *E. coli* and *K. pneumoniae*. Important differences were detected between the two degrees of protein binding. Only for strains with MIC of 0.25 mg/L and when low protein binding (50%) was taken into account, the PTAs reached in this analysis were >95%.

## Discussion

Overall, we found the PK/PD index of polymyxin B to best correlate with efficacy to be *f*AUC/MIC. Dose–response studies showed that stasis was achieved at *f*AUC/MIC values of 27.5–102.6 (median 63) for *E. coli* isolates and 5.9–60.5 (median 11.6) for *K. pneumoniae.* A 1 log reduction was only achieved in two *E. coli* isolates and one *K. pneumoniae* isolate.

The PK profile in mice showed non-linear PK with decreasing absorption rate and clearance with increasing doses. Non-linearity in clearance and absorption rate was already shown by Landersdorfer *et al.*,^8^ albeit described by a different function than in the current work (E_max_ versus power). Polymyxin B levels in ELF were very low, indicating low penetration in lungs after subcutaneous administration. These findings add to previous studies, in which subcutaneous administration to lung-infected mice did not result in killing of *K. pneumoniae* and *A. baumanni*^8,24^ and where relatively low polymyxin B levels were found in ELF.

Generally, *f*AUC/MIC is described as the best indicator for efficacy for other polymyxins.^7–9^ In our study, the *f*AUC/MIC inhibitory sigmoid dose–effect model showed a slightly better fit than the %*fT_>_*_MIC_ and *fC*_max_/MIC for *K. pneumoniae* 104. In contrast to *K. pneumoniae* 104, %*fT_>_*_MIC_ showed a marginally better fit than *f*AUC/MIC in the *E. coli* ATCC 25922 thigh infection model. Other studies where *f*AUC/MIC was reported as the assumed driver for efficacy also reported only small differences in goodness of fit of the different PK/PD indices.^5,8,10^

Stasis was found for all four *E. coli* and *K. pneumoniae* isolates, with *f*AUC/MIC targets for *E. coli* being higher than those for *K. pneumoniae* [median (range) 63 (27.5–102.6) versus 11.6 (5.9–60.5), respectively]. A 1 log kill was found only for 2/4 *E. coli* isolates in a range of 46.8–54.5 (median 50.6) and 1/4 *K. pneumoniae* isolate (25.2 *f*AUC/MIC). No 2 log kill effect was found for any isolate. When comparing *f*AUC/MIC targets, Landersdorfer *et al*.^8^ reported targets between 1.2 and 13.5 for a static effect and 3.7 and 28.0 for 1 log kill for three *K. pneumoniae* isolates. Our *f*AUC/MIC targets in four *K. pneumoniae* isolates fell in the range of 5.9–60.5 (median 11.6) for a static effect and was 25.2 for a 1 log reduction effect (one isolate). The differences in targets can partially be explained by differences found in unbound fraction. There is considerable inter-strain variation between target values found for the *f*AUC/MIC, especially for *K. pneumoniae*. This variation cannot be explained by differences between characteristics in bacterial growth. Overall, the *f*AUC/MIC targets for *E. coli* seem to be higher compared with *K. pneumoniae*. While both species belong to the Enterobacterales, it is known from urinary tract infections that there is a clinical difference between the two species, with *E. coli* causing more severe infections compared with *K. pneumoniae*.^25^ The difference in virulence might also explain the difference in values found in current study.

Protein binding of polymyxin B is known to be high. However, a difference was found between the current study (unbound fraction 20%) compared with previous studies (8.6% for polymyxin B,^8^ 8.4% for colistin^5^), which affects the PTA. In the present study, ultrafiltration was used for determining the unbound fraction, whilst ultracentrifugation was used in the other studies. With the ultrafiltration method, correction for the NSB to the ultrafiltration membranes was needed. In addition, differences in mouse plasma used for determination of polymyxin B protein binding may (partly) explain the discrepancy. Plasma of infected mice treated with polymyxin B was used, which likely contains polymyxin B metabolites as well, while spiked pooled plasma was used in the other studies. But instead of being methodology-related, differences in unbound fractions may rather reflect the difficulty of handling polymyxin B.

In clinical studies, the protein binding of polymyxin B is much higher than reported in mice, with protein binding in plasma up to 99% for polymyxin B.^22,26,27^ This applies to colistin as well.^5,28^ Therefore, the PK/PD targets based on the unbound fraction are necessary for translating PK/PD data of polymyxin B to the clinic.

To determine whether these targets are achieved in humans, the PTAs for the 200 mg q24h dose were calculated. It is clear that the degree of protein binding is essential herein and more clarity on the protein binding in human plasma is needed before an optimal dosing regimen can be recommended, since for a highly protein-bound drug a small error on the f_u_ would yield important PTA differences. Data on the WT distributions for polymyxin B are also lacking on the EUCAST website but MIC_90_ values in previous studies were ≤0.5–1 mg/L,^29^ which is higher than the lowest MIC 0.25 mg/L with PTA >95%. Furthermore, the 1 log kill effect, which is associated with clinical response in serious infections, was achieved in only 38% of isolates. EUCAST has currently no clinical breakpoints for polymyxin B; however, the United States Committee on Antimicrobial Susceptibility Testing (USCAST) has a susceptibility/resistance breakpoint for Enterobacterales of ≤2/≥4 mg/L. Given the data currently available, the targets might not be reached in all patients. This might contribute to the fact that polymyxin B has been associated with therapeutic failure previously.^30,31^

In conclusion, this study confirms that *f*AUC/MIC describes the exposure–response relationship for polymyxin B. The 1 log kill effect was achieved in the minority of the isolates whereas polymyxin B PK/PD targets cannot be attained for the majority of clinical isolates with the standard dosing regimen, indicating that polymyxin B monotherapy may be not effective against serious infections.

## Supplementary Material

dkad022_Supplementary_Data

## Supplementary data

Table S1 and Figures S1–S3 are available as Supplementary data at *JAC* Online.
