# Supplementary material for: Pharmacokinetic and pharmacodynamic properties of polymyxin B in Escherichia coli and Klebsiella pneumoniae murine infection models
Source: J Antimicrob Chemother. 2023 Jan 30;78(3):832–9. doi: 10.1093/jac/dkad022 (PMC10377753; doi:10.1093/jac/dkad022)
Supplement: dkad022_Supplementary_Data [file dkad022_supplementary_data.doc]

**Supplementary data**

**Table S1:** MIC characteristics for polymyxin B and resistance mechanisms of the *E. coli* and *K. pneumoniae* strains used in this study.

| **Species** | **Strain** | **Resistance summary (PCR)** | **MIC polymyxin B  (mg/L)** |
| --- | --- | --- | --- |
| *E. coli* | ATCC 25922 | No ESBL | 1 |
|  | 15 | CTX-M 15 | 1 |
|  | 51 | OXA-1, CTX-M 15 | 1 |
|  | 107 | VIM | 1 |
| *K. pneumoniae* | ATCC 43816 | No ESBL | 0.5 |
|  | 17 | SHV-1, OXA-1, CTX-M 15 | 1 |
|  | 58 | TEM 84, SHV 11 | 2 |
|  | 74 | CTX-M 15 | 2 |
|  | 104 | KPC-3, OmpK35red | 1 |


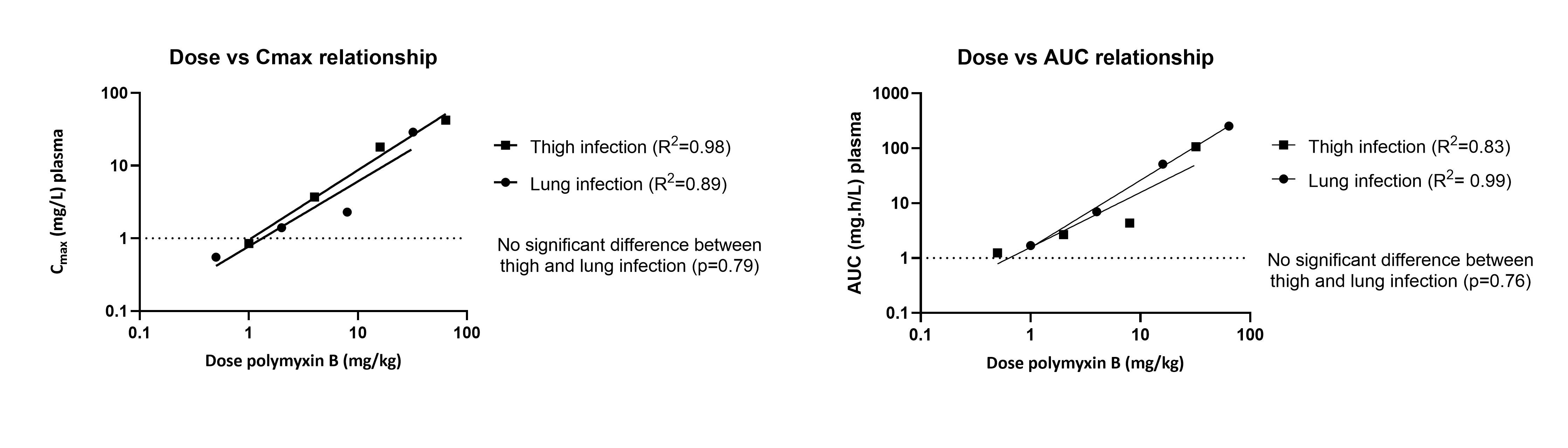


**Figure S1**. Dose-proportionality plot of polymyxin B after eight different subcutaneous doses of polymyxin B in mice, with four doses in the thigh infection model and four doses in the lung infection model. Each symbol represents the C_max_ or AUC per dose, R^2^ represents the goodness-of-fit.


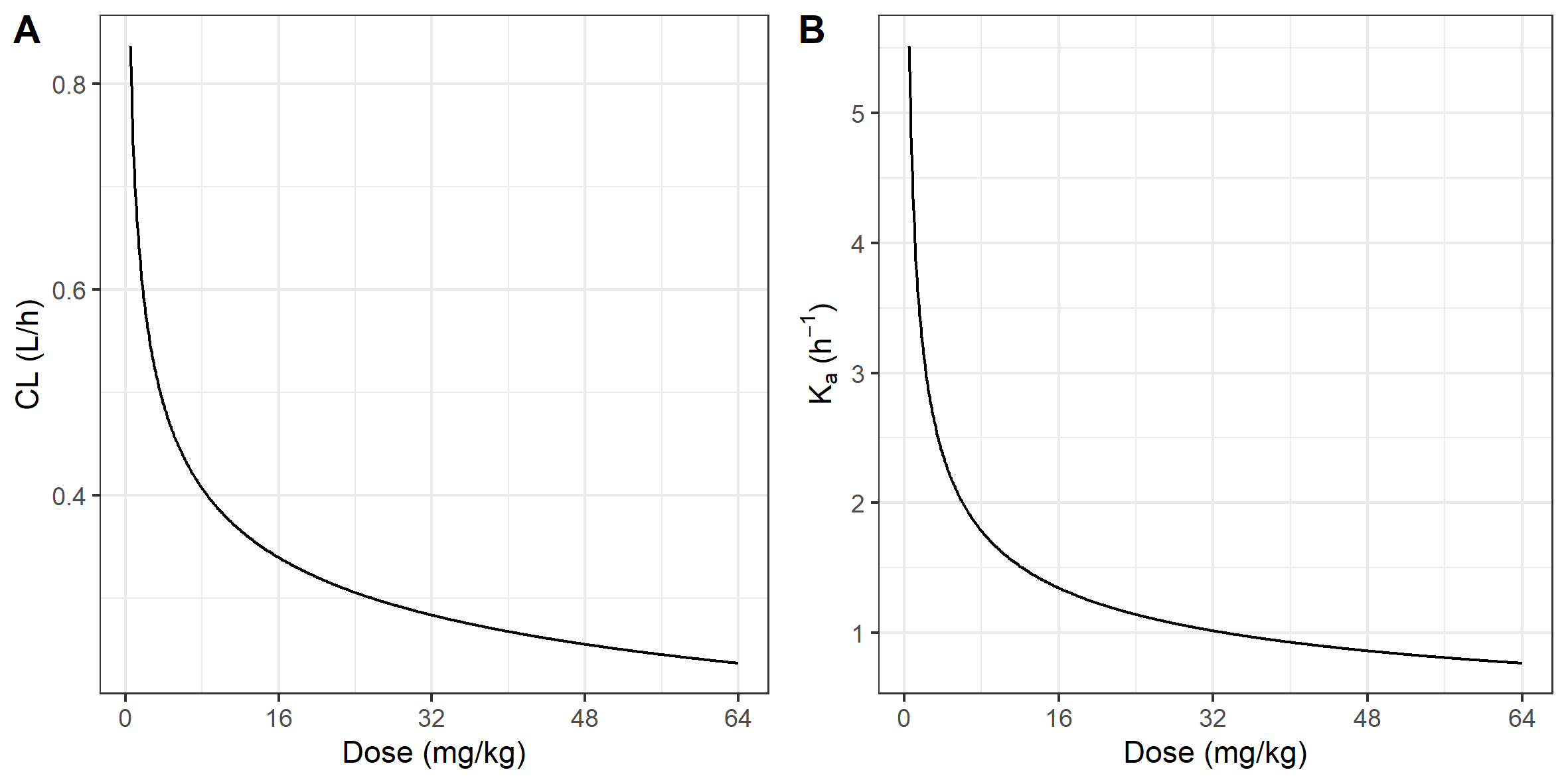


**Figure S2.** Graphical illustration of the dose dependency of A. Clearance and B. Absorption rate constant.

**
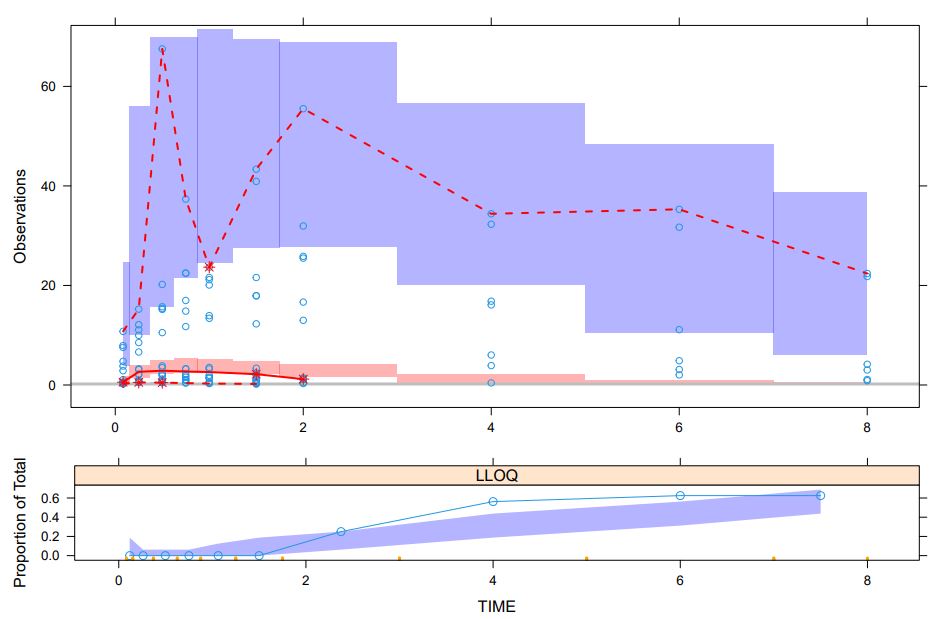
**

**Figure S3**. Visual predictive check (n=1,000) of the final model. Upper graph: blue dots represent observed polymyxin B concentrations, red lines indicate the mean and 95%CI of these observations. The shaded areas represent the mean (red) and 95%CI (blue) of the simulated data. The grey line indicates the LLOQ. Lower graph: blue dots show the proportion of observed concentrations <LOQ and the shaded area the simulated proportion of concentrations <LOQ.
